# Association between *BTLA* polymorphisms and susceptibility to esophageal squamous cell carcinoma in the Chinese population

**DOI:** 10.1002/jcla.23221

**Published:** 2020-02-15

**Authors:** Rui Cao, Weifeng Tang, Shuchen Chen

**Affiliations:** ^1^ Department of Thoracic Surgery Fujian Medical University Union Hospital Fuzhou China; ^2^ Department of Cardiothoracic Surgery Affiliated People's Hospital of Jiangsu University Zhenjiang China

**Keywords:** *BTLA*, ESCC, polymorphisms, susceptibility

## Abstract

**Background:**

Growing evidence suggested that B‐ and T‐lymphocyte attenuator (*BTLA*) polymorphisms raised the susceptibility to a wide range of cancers. This study aimed to evaluate whether *BTLA* variants were related to the risk of esophageal squamous cell carcinoma (ESCC).

**Methods:**

A total of 721 ESCC patients and 1208 matched non‐cancer controls were included in this research, and four tagging *BTLA* polymorphisms (rs2171513 G > A, rs3112270 A > G, rs1982809 G > A, and rs16859629 T > C) were selected and genotyped using SNPscan™ Assays.

**Results:**

In the present study, no significant relationship between *BTLA* polymorphisms and ESCC was observed. However, stratified analyses suggested that the variant of *BTLA* rs3112270 A > G reduced the risk of ESCC in the male subgroup (AG vs AA: adjusted OR = 0.78, 95% CI = 0.61‐0.99, *P* = .042), BMI < 24 kg/m^2^ subgroup (AG vs AA: adjusted OR = 0.72, 95% CI = 0.55‐0.93, *P* = .012; AG/GG vs AA: adjusted OR = 0.77, 95% CI = 0.60‐0.98, *P* = .032), and ever drinking subgroup (AG vs AA: adjusted OR = 0.61, 95% CI = 0.38‐0.97, *P* = .037). But when stratified by BMI ≥ 24 kg/m^2^, the rs3112270 A > G polymorphism increased the susceptibility to ESCC (GG vs AA: adjusted OR = 1.91, 95% CI = 1.02‐3.59, *P* = .045). Besides, we demonstrated that *BTLA* rs2171513 G > A polymorphism was protective of ESCC in the ever drinking subgroup (GA/AA vs GG: adjusted OR = 0.62, 95% CI = 0.39‐0.97, *P = *.037).

**Conclusion:**

Taken together, our initial investigation postulated that the rs3112270 A > G and rs2171513 G > A variants in the *BTLA* gene are candidates for the risk of ESCC, which might be helpful for the early diagnosis and treatment of ESCC.

## 1 INTRODUCTION

As stated by the global epidemiological data, esophageal cancer (EC) ranks the sixth primary cause of cancer‐related death, with an approximated 477 900 new occurrences and 375 000 deaths per year in China.^1^, ^2^ Different from the fact that esophagogastric junction adenocarcinoma (EGJA) is the dominant subtype of EC for the western nations, in China, esophageal squamous cell carcinoma (ESCC) makes up more than 90% of the total cases.^3^ And despite rapid progress in surgical technique and adjuvant treatment, the prognosis for patients with ESCC is extremely poor, with a 5‐year overall survival rate <30%.^4^ Thus, it is essential to explore new risk factors for further understanding the potential mechanism of ESCC progression.

Nowadays, the immune system plays an increasingly important role in anti‐tumor therapy.^5^ Cytotoxic T lymphocyte–associated antigen 4 (*CTLA‐4*) and programmed cell death 1 (*PD‐1*) are the prominent representative of this field. Similar to *CTLA‐4* and *PD‐1*, as a co‐inhibitory regulator of the immune system, *BTLA* contains an extracellular domain, a transmembrane region, and a cytoplasmic region.^6^ When combined with its ligand named herpesvirus entry mediator (*HVEM*),^7^ tyrosine phosphorylation of the cytoplasmic region in *BTLA* gene can suppress T‐cell activation by recruiting *Src* homology phosphatase‐1 and *Src* homology phosphatase‐2,^8^ which could significantly inhibit the secretion of *IL‐1, IFN‐γ*, and *IL‐10.* 9 And the role of *BTLA‐HVEM* pathway has also been identified in *BTLA*‐deficient mouse models,^10^ where the absence of *BTLA* gene could enhance sensitivity to antigen‐specific immune response and therefore develop autoimmune diseases, as well as *HVEM*‐deficient mice,^11^ which, on another level, showed the negative effect of *BTLA‐HVEM* pathway on the immune microenvironment.

In recent years, accumulating studies have focused on the genetic polymorphisms of immune molecules with susceptibility to the various tumors, including *BTLA*.^12^, ^13^, ^14^, ^15^ Fu et al^12^ genotyped five SNPs and found that *BTLA* rs1844089, rs2705535, and rs2633562 polymorphisms were associated with the pathological features of breast cancer. Partyka et al^13^ chose seven variants and revealed the rs1982809G allele contributed to a higher‐grade stage of renal cell carcinoma. Recently, there was a basic study demonstrating that the change from T to C in the *BTLA* rs1982809 variant could interfere with the activity of *BTLA* 3'UTR and regulate *BTLA* expression in peripheral blood T lymphocytes, which might be considered as a potential biomarker in predicting the process of sepsis and multiple organ dysfunction syndrome.^16^ In addition, Karabon et al^14^ enrolled a total of ten polymorphisms and demonstrated that the presence of *BTLA* rs1982809 polymorphism was related to a lower level of *BTLA* mRNA, and the variant might be deemed as a low‐risk factor for the development of chronic lymphocytic leukemia. Subsequently, Tang et al^15^ reported that the *BTLA* rs1982809 SNP was found to be conferred to an increased risk of EGJA in smoking patients.

Nevertheless, whether the variation in the *BTLA* gene associates with ESCC risk remains unknown. Concerning the tremendous value of co‐signaling molecules in anti‐tumor therapy, and to better understand this issue, we conducted this case‐control study to clarify the detailed relationship of four tagging *BTLA* polymorphisms with the risk of ESCC in the eastern Chinese Han population.

## 2 MATERIALS AND METHODS

### 2.1 Ethics statement

All procedures of this research were administered in line with the Declaration of Helsinki and approved by the Institutional Review Board of Jiangsu University (NO. K‐20160036‐Y). Each participant provided the written informed consent to this study and was willing to donate 2 mL of peripheral blood.

### 2.2 Participants

From February 2014 to April 2018, patients with pathologically confirmed ESCC were continuously recruited from Fujian Medical University Union Hospital and the Affiliated People's Hospital of Jiangsu University. The major exclusion criteria for ESCC subjects were as follows: (a) suffering from autoimmune diseases, (b) prior exposure to anti‐cancer treatment, (c) history of any other malignancy, and (d) patients with incomplete clinical records. Ultimately, 721 ESCC cases were enrolled in this study. During the parallel period, 1208 healthy controls were also recruited from the department of physical examination in the same hospitals and matched with the ESCC patients in terms of age and sex. And the control individuals should meet the major inclusion criteria: (a) non‐cancer samples, (b) without any infectious/immunological disorders, and (c) ethnicity of the eastern Chinese Han population. The detailed data on personal characteristics and environmental factors, including smoking status and alcohol consumption, were obtained by questionnaires and patients' clinical records. We defined the “ever drinkers” as the subjects with drinking no <3 times a week for longer than half a year, and the individuals who smoked at least one cigarette per day over 1 year were deemed as “ever smokers.” Besides, we used the body mass index (BMI) value of 24 kg/m^2^ as a threshold for distinguishing individuals at obesity.^17^

### 2.3 SNP selection

*BTLA* tagging SNPs were ascertained based on the Genome Variation Server data (http://gvs.gs.washington.edu/GVS147/), with the extent covering all the gene regions together with the upstream and downstream extending 5 Kb, respectively. And the following criteria were applied: minor allele frequency (MAF) ≥0.05 and minimum linkage disequilibrium (LD) of *r* ^2^ < .8. Overall, four candidate *BTLA* SNPs, including rs2171513 G > A, rs3112270 A > G, rs1982809 G > A, and rs16859629 T > C, were enrolled in this research to evaluate the effect of *BTLA* polymorphisms on the susceptibility to ESCC.

### 2.4 DNA genotyping

The whole blood sample was stored in an anti‐coagulated tube that contained EDTA. We extracted the genomic DNA by using a Promega DNA Mini Kit (Promega) under the instruction of the manufacturer's procedure,^18^ and then, the four SNPs were genotyped using the SNPscan™ Assays (Genesky Biotechnologies Inc).^19^ For qualitative tests, 4% of the total DNA samples were selected at random and genotyped again by different laboratory staff, and the final results of the four *BTLA* genotypes were in concord with the primary findings.

### 2.5 Statistical analysis

In this study, all data analyses were conducted with software SAS version 9.4 (SAS Institute). The value of continuous variable, including age, was reported as means ± standard deviation (SD) and evaluated by Student's *t* test. The comparison of categorical variables between ESCC cases and controls, such as *BTLA* genotypes, was conducted with the chi‐square test. The deviation of HWE for each SNP distribution in the controls was assessed via the online software (http://ihg.gsf.de/cgi-bin/hw/hwa1.pl). After adjusting for age, gender, smoking status, and alcohol consumption, the potential associations between *BTLA* variants and the risk of ESCC were examined by the multivariate logistic regression analyses and described by calculating the adjusted odds ratio (OR) with 95% confidence intervals (CIs). A two‐sided *P* value < .05 was deemed as statistically significant.

## 3 RESULTS

### 3.1 Basic characteristics

Basic information regarding *BTLA* polymorphisms is revealed in Table 1. Results showed that the MAF of each *BTLA* SNP was in accord with the database of the Chinese population. In the control group, frequencies of the four *BTLA* genotypes were all reached HWE (all *P* > .05), and the failed genotype data for each polymorphism were <1%. Table 2 summarizes the basic features of 721 ESCC cases and 1208 controls, and the mean age of the case and control groups was 62.59 ± 8.18 and 62.92 ± 8.94 years, respectively. The ESCC group composed of 551 males (76.42%) and 170 females (23.58%), and there involved 899 males (74.42%) and 309 females (25.58%) among the controls. There was no difference in age and sex between the study groups (both *P* > .05), meaning that the two above factors were well matched. However, compared with the controls, the degree of BMI and the proportion of drinking and smoking were significantly higher in those of ESCC group (all *P* < .05).

**Table 1:** Primary information for *BTLA* tagging SNPs

Genotyped polymorphisms	rs2171513 G > A	rs3112270 A > G	rs1982809 G > A	rs16859629 T > C
Chr	3	3	3	3
Position_38	112466080	112461797	112463893	112471533
Region	3'UTR	Promoter	3'UTR	intron_variant
MAF in database (1000 genomes‐ Chinese Han populations)	0.188	0.269	0.216	0.067
MAF in our controls (n = 1208)	0.197	0.281	0.260	0.081
*P* value for HWE test in our controls	.551	.026	.108	.958
% Genotyping value	99.27%	99.12%	99.22%	99.29%

Abbreviations: HWE, Hardy‐Weinberg equilibrium; MAF, minor allele frequency.

**Table 2:** Distribution of selected demographic variables and risk factors in ESCC cases and controls

Variable	Cases (n = 721)	Controls (n = 1208)	*P*
	n (%)	n (%)	
Age (years)	62.59 ± 8.18	62.92 ± 8.94	.413
Age (years)			
<63	337 (46.74)	579 (47.93)	.613
≥63	384 (53.26)	629 (52.07)	
Sex			
Male	551 (76.42)	899 (74.42)	.325
Female	170 (23.58)	309 (25.58)	
Tobacco use			
Never	342 (47.43)	881 (72.93)	**<.001**
Ever	379 (52.57)	327 (27.07)	
Alcohol use			
Never	502 (69.63)	1046 (86.59)	**<.001**
Ever	219 (30.37)	162 (13.41)	
BMI (kg/m^2^)			
<24	527 (73.09)	651 (53.89)	**<.001**
≥24	194 (26.01)	557 (46.11)	

Note

Bold values are statistically significant (*P* < .05).

Abbreviation: BMI, body mass index.

### 3.2 *BTLA* polymorphisms and ESCC risk in the overall population

The detailed frequencies of *BTLA* genotypes and the results about the association between each selected polymorphism with the risk of ESCC are presented in Table 3. We found that *BTLA* rs2171513 G > A, rs3112270 A > G, and rs1982809 G > A SNPs were not correlated with the susceptibility to the entire cohorts (all *P* > .05). Nevertheless, we showed that the *BTLA* rs16859629 T > C variant significantly decreased the risk of ESCC (TC vs TT: adjusted OR = 0.75, 95% CI = 0.57‐0.99, *P* = .044; TC/CC vs TT: adjusted OR = 0.75, 95% CI = 0.57‐0.98, *P* = .035). But the significant statistical distribution of *BTLA* rs16859629 T > C SNP disappeared after adjusting for the confounding factors, including age, sex, smoking, and alcohol status (*P* > .05).

**Table 3:** Genotype frequencies of *BTLA* tagging SNPs and ESCC risk

Genotype	ESCC cases (n = 721)		Controls (n = 1208)		Crude OR (95% CI)	*P*	Adjusted OR (95% CI)^a^	*P*
	n	%	N	%				
rs2171513 G > A								
GG	463	64.85	774	64.45	1.00		1.00	
GA	227	31.79	380	31.64	1.00 (0.82‐1.22)	.989	0.99 (0.80‐1.22)	.888
AA	24	3.36	47	3.91	0.85 (0.52‐1.42)	.539	0.80 (0.47‐1.37)	.424
GA + AA	251	35.15	427	35.55	0.98 (0.81‐1.19)	.860	0.96 (0.79‐1.19)	.730
GG + GA	690	96.64	1154	96.09	0.85 (0.52‐1.41)	.537	0.81 (0.47‐1.37)	.430
A allele	275	19.26	474	19.73				
rs3112270 A > G								
AA	387	54.43	614	51.12	1.00		1.00	
AG	259	36.43	500	41.64	0.82 (0.68‐1.00)	.051	0.84 (0.68‐1.04)	.109
GG	65	9.14	87	7.24	1.19 (0.84‐1.68)	.335	1.27 (0.88‐1.83)	.204
AG + GG	324	45.57	587	48.88	0.88 (0.73‐1.06)	.162	0.91 (0.74‐1.10)	.324
AA + AG	646	90.86	1114	92.76	1.29 (0.92‐1.80)	.139	1.36 (0.96‐1.95)	.087
G allele	389	27.36	674	28.06				
rs1982809 G > A								
GG	408	57.22	657	54.70	1.00		1.00	
GA	252	35.35	464	38.64	0.88 (0.72‐1.07)	.182	0.93 (0.75‐1.15)	.488
AA	53	7.43	80	6.66	1.07 (0.74‐1.54)	.731	1.30 (0.88‐1.91)	.189
GA + AA	305	42.78	544	45.30	0.90 (0.75‐1.09)	.284	0.98 (0.80‐1.20)	.841
GG + GA	660	92.57	1121	93.34	1.13 (0.79‐1.61)	.521	1.34 (0.92‐1.95)	.134
A allele	358	25.11	624	25.98				
rs16859629 T > C								
TT	622	87.61	997	84.06	1.00		1.00	
TC	85	11.97	181	15.27	**0.75 (0.57‐0.99)**	**.044**	0.82 (0.61‐1.10)	.191
CC	3	0.42	8	0.67	0.60 (0.16‐2.28)	.454	0.82 (0.21‐3.23)	.777
CT + CC	88	12.39	189	15.94	**0.75 (0.57‐0.98)**	**.035**	0.82 (0.62‐1.10)	.184
TT + CT	707	99.58	1178	99.33	0.63 (0.17‐2.36)	.489	0.85 (0.22‐3.33)	.809
C allele	91	6.31	197	8.15				

Note

Bold values are statistically significant (*P* < .05).

Abbreviations: CI, confidence interval; OR, odds ratio.

a Adjusted for age, sex, smoking status, and alcohol use in a logistic regression model.

### 3.3 *BTLA* polymorphisms and ESCC risk in stratification groups

Furthermore, we conducted a stratified analysis mainly relied on the enrolled parameters, including age, sex, BMI, smoking status, and alcohol consumption. As presented in Table 4, when stratified by alcoholic use, in the ever drinking subgroup, we found that the rs2171513 G > A variant in *BTLA* gene might be a protective variable against the progression of ESCC (GA/AA vs GG: adjusted OR = 0.62, 95% CI = 0.39‐0.97, *P* = .037).

**Table 4:** Stratified analyses between *BTLA* rs2171513 G > A polymorphism and ESCC risk by sex, age, smoking status, and alcohol consumption

Variable	*BTLA* rs2171513 G > A (case/control)^a^			Adjusted OR (95% CI)^b^; *P*				
	GG	GA	AA	GG	GA vs GG	AA vs GG	GA/AA vs GG	AA vs (GG/GA)
Sex								
Male	352/579	175/278	18/38	1.00	1.01 (0.78‐1.29); *P*: .966	0.73 (0.40‐1.36); *P*: .322	0.97 (0.77‐1.24); *P*: .816	0.73 (0.40‐1.35); *P*: .315
Female	111/195	52/102	6/9	1.00	0.91 (0.60‐1.38); *P*: .653	1.04 (0.34‐3.17); *P*: .945	0.92 (0.61‐1.38); *P*: .685	1.07 (0.36‐3.24); *P*: .899
Age								
<63	219/578	103/177	11/22	1.00	0.97 (0.70‐1.34); *P*: .860	0.76 (0.34‐1.71); *P*: .510	0.95 (0.69‐1.29); *P*: .731	0.77 (0.35‐1.72); *P*: .522
≥63	244/396	124/203	13/25	1.00	1.00 (0.75‐1.33); *P*: .985	0.80 (0.39‐1.64); *P*: .535	0.98 (0.74‐1.28); *P*: .855	0.80 (0.39‐1.63); *P*: .533
Smoking status								
Never	229/562	98/281	12/31	1.00	0.85 (0.64‐1.13); *P*: .264	1. (0.49‐2.02); *P*: .996	0.87 (0.66‐1.14); *P*: .297	1.05 (0.52‐2.11); *P*: .889
Ever	234/212	129/99	12/16	1.00	1.21 (0.87‐1.68); *P*: .270	0.62 (0.28‐1.36); *P*: .233	1.12 (0.81‐1.54); *P*: .489	0.58 (0.27‐1.27); *P*: .173
Alcohol consumption								
Never	315/678	163/323	20/39	1.00	1.08 (0.85‐1.38); *P*: .513	0.96 (0.54‐1.73); *P*: .895	1.07 (0.85‐1.35); *P*: .569	0.94 (0.53‐1.67); *P*: .824
Ever	148/96	64/57	4/8	1.00	0.66 (0.41‐1.05); *P*: .082	0.31 (0.08‐1.13); *P*: .077	**0.62 (0.39‐0.97);** ***P*: .037**	0.35 (0.10‐1.29); *P*: .115
BMI (kg/m^2^)								
<24	336/420	167/196	17/29	1.00	1.03 (0.79‐1.35); *P*: .811	0.66 (0.35‐1.25); *P*: .203	0.98 (0.76‐1.27); *P*: .891	0.65 (0.35‐1.23); *P*: .188
≥24	127/354	60/184	7/18	1.00	0.89 (0.62‐1.28); *P*: .521	1.35 (0.54‐3.40); *P*: .522	0.92 (0.65‐1.31); *P*: .656	1.40 (0.56‐3.51); *P*: .467

Note

Bold values are statistically significant (*P* < .05).

Abbreviations: BMI, body mass index; CI, confidence interval; OR, odds ratio.

a The genotyping was successful in 714 (99.03%) ESCC cases and 1201 (99.42%) controls for *BTLA* rs2171513 G > A.

b Adjusted for age, sex, smoking status, and alcohol consumption (besides stratified factors accordingly) in a logistic regression model.

As exhibited in Table 5, there was a close correlation between *BTLA* rs3112270 A > G and the risk of ESCC in some certain subgroups. In the male population, results demonstrated that the genotype of AG in *BTLA* rs3112270 lowered the ESCC risk when using AA genotype as a reference (AG vs AA: adjusted OR = 0.78, 95% CI = 0.61‐0.99, *P* = .042). And in ever drinking subgroup, we found a similar unfavorable effect of AG genotype on the risk of ESCC (AG vs AA: adjusted OR = 0.61, 95% CI = 0.38‐0.97, *P* = .037). When stratified by BMI, analyses showed that the rs3112270 A > G variant decreased the genetic susceptibility to ESCC in the BMI < 24 kg/m^2^ subgroup (AG vs AA: adjusted OR = 0.72, 95% CI = 0.55‐0.93, *P* = .012; AG/GG vs AA: adjusted OR = 0.77, 95% CI = 0.60‐0.98, *P* = .032). But in the BMI ≥ 24 kg/m^2^ population, the outcome of this SNP conferred an opposite effect on the development of ESCC (GG vs AA: adjusted OR = 1.91, 95% CI = 1.02‐3.59, *P* = .045).

**Table 5:** Stratified analyses between *BTLA* rs3112270 *A > G* polymorphism and ESCC risk by sex, age, smoking status, and alcohol consumption

Variable	*BTLA* rs3112270 A > G (case/control)^a^			Adjusted OR (95% CI)^b^; *P*				
	AA	GA	GG	AA	AG vs AA	GG vs AA	AG/ GG vs AA	GG vs (AA/AG)
Sex								
Male	305/459	190/372	48/64	1.00	**0.78 (0.61‐0.99);** ***P*: .042**	1.18 (0.77‐1.81); *P*: .459	0.83 (0.66‐1.05); *P*: .123	1.31 (0.86‐1.99); *P*: .209
Female	82/155	69/128	17/23	1.00	1.08 (0.72‐1.62); *P*: .720	1.66 (0.82‐3.36); *P*: .158	1.16 (0.79‐1.71); *P*: .451	1.61 (0.82‐3.16); *P*: .172
Age								
<63	180/291	118/239	33/47	1.00	0.77 (0.56‐1.06); *P*: .111	1.19 (0.71‐2.01); *P*: .514	0.84 (0.63‐1.13); *P*: .256	1.32 (0.80‐2.20); *P*: .280
≥63	207/323	141/261	32/40	1.00	0.88 (0.66‐1.16); *P*: .357	1.33 (0.79‐2.23); *P*: .281	0.94 (0.72‐1.22); *P*: .624	1.41 (0.85‐2.33); *P*: .183
Smoking status								
Never	179/446	124/361	34/67	1.00	0.86 (0.66‐1.14); *P*: .295	1.27 (0.80‐2.01); *P*: .306	0.93 (0.72‐1.20); *P*: .572	1.35 (0.87‐2.11); *P*: .183
Ever	208/168	135/139	31/20	1.00	0.80 (0.58‐1.10); *P*: .169	1.33 (0.72‐2.47); *P*: .366	0.86 (0.631‐1.17); *P*: .348	1.46 (0.80‐2.68); *P*: .217
Alcohol consumption								
Never	259/530	189/432	48/78	1.00	0.90 (0.71‐1.14); *P*: .388	1.28 (0.86‐1.92); *P*: .225	0.96 (0.77‐1.20); *P*: .720	1.34 (0.91‐1.99); *P*: .139
Ever	128/84	70/68	17/9	1.00	**0.61 (0.38‐0.97);** ***P*: .037**	1.25 (0.49‐3.21); *P*: .643	0.68 (0.44‐1.06); *P*: .087	1.52 (0.61‐3.82); *P*: .373
BMI (kg/m^2^)								
<24	296/325	176/267	46/53	1.00	**0.72 (0.55‐0.93);** ***P*: .012**	1.03 (0.66‐1.61); *P*: .906	**0.77 (0.60‐0.98);** ***P*: .032**	1.18 (0.76‐1.82); *P*: .462
≥24	91/289	83/233	19/34	1.00	1.16 (0.81‐1.65); *P*: .420	**1.91 (1.02‐3.59);** ***P*: .045**	1.25 (0.897‐1.76); *P*: .200	1.78 (0.97‐3.27); *P*: .062

Note

Bold values are statistically significant (*P* < .05).

Abbreviations: BMI, body mass index; CI, confidence interval; OR, odds ratio.

a The genotyping was successful in 711 (98.61%) ESCC cases and 1201 (99.42%) controls for *BTLA* rs3112270 A > G.

b Adjusted for age, sex, smoking status, and alcohol consumption (besides stratified factors accordingly) in a logistic regression model.

However, as shown in Tables 6 and 7, our results identified that there was no significant difference of distribution in *BTLA* rs1982809 G > A and rs16859629 T > C variants among any ESCC subgroup and age‐/sex‐matched controls (all *P* > .05).

**Table 6:** Stratified analyses between *BTLA* rs1982809 G > A polymorphism and ESCC risk by sex, age, smoking status, and alcohol consumption

Variable	*BTLA* rs1982809 G > A (case/control)^a^			Adjusted OR (95% CI)^b^; *P*				
	GG	GA	AA	GG	GA vs GG	AA vs GG	GA/AA vs GG	AA vs (GG/GA)
Sex								
Male	316/497	189/342	39/56	1.00	0.95 (0.74‐1.21); *P*: .669	1.37 (0.87‐2.17); *P*: .178	1.01 (0.80‐1.27); *P*: .967	1.40 (0.89‐2.20); *P*: .142
Female	92/160	63/122	14/24	1.00	0.89 (0.60‐1.34); *P*: .586	1.20 (0.58‐2.49); *P*: .626	0.94 (0.64‐1.38); *P*: .747	1.26 (0.62‐2.55); *P*: .528
Age								
<63	192/302	111/232	30/43	1.00	0.78 (0.57‐1.07); *P*: .119	1.36 (0.80‐2.34); *P*: .260	0.86 (0.64‐1.16); *P*: .332	1.51 (0.89‐2.55); *P*: .125
≥63	216/355	141/232	23/37	1.00	1.05 (0.80‐1.40); *P*: .718	1.18 (0.67‐2.07); *P*: .567	1.07 (0.82‐1.40); *P*: .623	1.16 (0.66‐2.01); *P*: .610
Smoking status								
Never	186/470	121/339	32/65	1.00	0.91 (0.69‐1.19); *P*: .483	1.25 (0.79‐2.00); *P*: .344	0.96 (0.74‐1.25); *P*: .772	1.30 (0.83‐2.05); *P*: .252
Ever	222/187	131/125	21/15	1.00	0.96 (0.69‐1.33); *P*: .802	1.46 (0.71‐2.97); *P*: .304	1.01 (0.74‐1.38); *P*: .954	1.48 (0.73‐2.99); *P*: .275
Alcohol consumption								
Never	270/558	189/410	39/72	1.00	0.96 (0.76‐1.22); *P*: .753	1.22 (0.79‐1.88); *P*: .364	1.00 (0.80‐1.25); *P*: .998	1.24 (0.82‐1.88); *P*: .315
Ever	138/99	63/54	14/8	1.00	0.76 (0.47‐1.23); *P*: .270	1.98 (0.72‐5.45); *P*: .188	0.88 (0.56‐1.38); *P*: .576	2.16 (0.80‐5.89); *P*: .131
BMI (kg/m^2^)								
<24	306/355	180/245	33/45	1.00	0.88 (0.68‐1.14); *P*: .329	1.11 (0.67‐1.82); *P*: .695	0.91 (0.71‐1.16); *P*: .454	1.16 (0.72‐1.89); *P*: .545
≥24	102/302	72/219	20/35	1.00	1.02 (0.71‐1.46); *P*: .936	1.71 (0.93‐3.16); *P*: .087	1.11 (0.79‐1.57); *P*: .532	1.70 (0.94‐3.08); *P*: .081

Abbreviations: BMI, body mass index; CI, confidence interval; OR, odds ratio.

a The genotyping was successful in 713 (98.89%) ESCC cases and 1201 (99.42%) controls for *BTLA* rs1982809 G > A.

b Adjusted for age, sex, smoking status, and alcohol consumption (besides stratified factors accordingly) in a logistic regression model.

**Table 7:** Stratified analyses between *BTLA* rs16859629 T > C polymorphism and ESCC risk by sex, age, smoking status, and alcohol consumption

Variable	*BTLA* rs16859629 T > C (case/control)^a^			Adjusted OR (95% CI)^b^; *P*				
	TT	TC	CC	TT	TC vs TT	CC vs TT	TC/CC vs TT	CC vs (TT/TC)
Sex								
Male	469/739	68/138	3/5	1.00	0.87 (0.63‐1.22); *P*: .433	1.73 (0.39‐7.65); *P*: .469	0.90 (0.65‐1.25); *P*: .519	1.77 (0.40‐7.82); *P*: .451
Female	153/258	17/43	0/3	1.00	0.68 (0.37‐1.25); *P*: .213	‐	0.63 (0.34‐1.14); *P*: .125	‐
Age								
<63	295/481	37/85	0/3	1.00	0.79 (0.50‐1.24); *P*: .305	‐	0.75 (0.48‐1.18); *P*: .215	‐
≥63	327/516	48/96	3/5	1.00	0.85 (0.57‐1.25); *P*: .398	1.41 (0.32‐6.21); *P*: .649	0.87 (0.59‐1.27); *P*: .466	1.45 (0.33‐6.38); *P*: .623
Smoking status								
Never	292/716	42/143	3/7	1.00	0.74 (0.51‐1.08); *P*: .116	0.97 (0.24‐3.92); *P*: .968	0.75 (0.52‐1.08); *P*: .126	1.02 (0.25‐4.10); *P*: .981
Ever	330/281	43/38	0/1	1.00	0.98 (0.61‐1.58); *P*: .931	‐	0.95 (0.59‐1.54); *P*: .847	‐
Alcohol consumption								
Never	436/863	58/157	3/7	1.00	0.78 (0.56‐1.09); *P*: .145	0.99 (0.25‐3.99); *P*: .988	0.79 (0.57‐1.09); *P*: .154	1.03 (0.26‐4.14); *P*: .971
Ever	186/134	27/24	0/1	1.00	1.00 (0.52‐1.90); *P*: .993	‐	0.95 (0.50‐1.80); *P*: .879	‐
BMI (kg/m^2^)								
<24	455/534	61/96	2/5	1.00	0.82 (0.57‐1.17); *P*: .275	0.75 (0.14‐3.97); *P*: .734	0.82 (0.57‐1.16); *P*: .259	0.77 (0.15‐4.09); *P*: .761
≥24	167/463	24/85	1/3	1.00	0.84 (0.51‐1.39); *P*: .508	1.07 (0.10‐11.24); *P*: .958	0.85 (0.52‐1.39); *P*: .522	1.09 (0.10‐11.53); *P*: .941

Abbreviations: BMI, body mass index; CI, confidence interval; OR, odds ratio.

a The genotyping was successful in 710 (98.47%) ESCC cases and 1186 (98.18%) controls for *BTLA* rs16859629 T > C.

b Adjusted for age, sex, smoking status, and alcohol consumption (besides stratified factors accordingly) in a logistic regression model.

## 4 DISCUSSION

Recently, increasing evidence has identified the role of immunosurveillance in supporting tumor growth, and various checkpoint inhibitors, such as ipilimumab and pembrolizumab, which represent the *CTLA‐4* and *PD‐1* molecules, have been proven as successful in some iconic clinical trials that refer to the treatment of several advanced tumors.^20^, ^21^ And as related to the complicated cause of ESCC, although undefined, multiple gene loci have been confirmed to drive esophageal lesions, which led to a more poor prognosis of ESCC.^22^ In this case‐control study of exploring the potential association between polymorphisms of the co‐inhibitory *BTLA* gene with the susceptibility to ESCC, we found those four tagging SNPs might not influence the entire ESCC risk for the first time. But stratified analyses found a significant relationship between the two candidate SNPs of rs3112270 A > G and rs2171513 G > A and ESCC risk, which indicated the two polymorphisms in *BTLA* gene might be involved in the etiology of ESCC.

The pathogenesis of ESCC is complex, where the interrelationship between environmental exposures and individual genetic mutations could result in the deterioration of ESCC.^23^, ^24^, ^25^ As revealed in our study, there were possible gene‐environment interactions for the polymorphisms of *BTLA* rs3112270 A > G with ESCC susceptibility; especially for the individuals with different BMI settings, their corresponding risk of ESCC was different. Although the mechanism between BMI and ESCC development remained unclear, and concerning that BMI could reflect the status of body nutrition, there was some possible evidence proving that obesity was correlated with the increased level of cancer‐related hormones, such as insulin‐like growth factor, which could be involved in the regulation of cell cycle.^26^ Additionally, the site of this SNP was located at the promoter region of *BTLA* gene, where this region could bind to some proteins and further affect the process of DNA transcription and translation in vitro,^27^, ^28^ which might explain the mutation from A to G in *BTLA* gene could influence the progression of ESCC. Considering the role of this SNP was not set up yet, more case‐control studies should be conducted to clarify the accurate mechanism of this variant.

As for the rs2171513 G > A polymorphism, we identified a significant difference in the distribution of *BTLA* rs2171513 G > A variant in the ever drinking subgroup, which suggested the frequencies of GG genotype in rs2171513 are higher in ESCC subjects than that of the controls, which was consistent with previous researches.^29^, ^30^ Yang et al^29^ showed that the AA and GA genotypes of this SNP were associated with increased susceptibility to ankylosing spondylitis among the Chinese population, while Lnuo et al^30^ found no distribution differences in alleles, genotypes, and haplotypes of this SNP for type 1 diabetes and systemic lupus erythematosus among Japanese people. Besides, the expression of *BTLA* was found to be up‐regulated in various tumors.^31^, ^32^ For instance, Oguro et al^31^ showed that the elevated expression of *BTLA* was closely correlated with a lower density of CD8^+^ T cells, and further indicated that the higher expression of *BTLA* was associated with a worse prognosis in gallbladder cancer patients. Taken together, we postulated that the genetic mutation occurred in the exon 5 of *BTLA* gene might influence the function of *BTLA‐HVEM* pathway, where this signaling pathway has been proven to decrease local immune response in the tumor tissue of ESCC.^33^ In the future, more replicated studies about this SNP are required to confirm our hypothesis in esophageal carcinogenesis.

However, several limitations should be addressed when explaining the final results. First, the included participants with limited samples were originated from only two hospitals, which could not fully represent the eastern Chinese population and might inevitably lead to selection bias. So we used stratified analyses as compensation. Second, only four tagging *BTLA* SNPs were selected in this study, which might restrict to draw a firm conclusion about the exact relationship of *BTLA* polymorphisms with the risk of ESCC. Third, despite the samples of our research were relatively large, unfortunately, we had no extra DNA specimens to validate our primary findings. Finally, in the current research, functional experiments were not carried out to explore the biologic mechanisms of these polymorphisms during the development of ESCC.

## 5 CONCLUSION

Despite these limitations, our preliminary findings suggest that the two tagging variants of rs3112270 A > G and rs2171513 G > A in the *BTLA* gene might contribute to the progression of ESCC in the eastern Chinese population, which is the first study for the involvement of the co‐inhibitory *BTLA* SNPs in ESCC to our knowledge. But future intensive studies with larger samples are worth to elucidate these works as well as the underlying molecular function of *BTLA* polymorphisms.

## CONFLICT OF INTEREST

The author reports no potential financial conflicts of interest in this work.
